# Serum N^1^-methylnicotinamide is Associated with Left Ventricular Systolic Dysfunction in Chinese

**DOI:** 10.1038/s41598-018-26956-7

**Published:** 2018-06-05

**Authors:** Ming Liu, Anxia He, Jihong Chu, Chao Chen, Siqi Zhang, Yun He, Weiwei Tao, Meijuan Lu, Mulian Hua, Wenzheng Ju, Zhuyuan Fang

**Affiliations:** 1 Institute of Hypertension, Jiangsu Province Hospital of Traditional Chinese Medicine, The Affiliated Hospital of Nanjing University of Chinese Medicine, Nanjing, Jiangsu China; 2 Department of Cardiology, Jiangsu Province Hospital of Traditional Chinese Medicine, The Affiliated Hospital of Nanjing University of Chinese Medicine, Nanjing, Jiangsu China; 3 Department of Echocardiography, Jiangsu Province Hospital of Traditional Chinese Medicine, The Affiliated Hospital of Nanjing University of Chinese Medicine, Nanjing, Jiangsu China; 4 Department of Clinical Pharmacology, Jiangsu Province Hospital of Traditional Chinese Medicine, The Affiliated Hospital of Nanjing University of Chinese Medicine, Nanjing, Jiangsu China

## Abstract

We previously reported that serum N^1^-methylnicotinamide (me-NAM), an indicator of nicotinamide N-methyltransferase (NNMT) activity, was associated with obesity, diabetes, and coronary artery disease in Chinese patients. However, whether NNMT might play a role in the development of heart failure remains to be elucidated. In this study, the associations between levels of serum me-NAM and left ventricular structure and function were investigated in Chinese patients. Serum me-NAM was measured by liquid chromatography-mass spectrometry in 265 subjects. M-mode, 2-dimensional and Doppler echocardiography were performed with the GE Vivid E9 system to assess left ventricular structure and function. Of note, the participants in the top tertile of me-NAM had the lowest left ventricular ejection fraction (LVEF), preload recruitable stroke work (PRSW), and highest prevalence of left ventricular systolic dysfunction (LVSD). Serum me-NAM was negatively correlated with LVEF and PRSW before and after adjusted for potential confounding variables (*P* ≤ 0.02). In multiple logistic regression analyses, the subjects in the top tertile of me-NAM had highest risks for LVSD (OR 6.80; 95% CI 1.26–36.72; *P* = 0.026), which was also observed in continuous analyses (OR 9.48; 95% CI 1.41–63.48; *P* = 0.02). In conclusion, serum me-NAM is negatively associated with LVEF and PRSW and accordingly positively associated with the prevalence of LVSD in Chinese patients.

## Introduction

Congestive heart failure (HF), a leading cause of morbidity and mortality, continues to be a major public health problem^1^. Despite some progress, most HF patients experience distressing symptom that lead poor quality of life and a high incidence of rehospitalization^2^. In addition to structural remodeling, remodeling of cardiac energy metabolism can contribute to the severity of chronic HF^3^. However, the underlying mechanism between energy metabolism and chronic HF has not yet been clarified.

It was recently revealed that nicotinamide N-methyltransferase (NNMT) is a novel modulator of histone methylation and plays an important role in regulating cellular energy metabolism via NAD^+^ and polyamine flux pathways in animal studies^4^. N^1^-methylnicotinamide (me-NAM) has been used as an indicator of NNMT activity because it can only be derived from the methylation reaction catabolized by NNMT in the liver^5^. Me-NAM was initially considered as an inactive metabolite of terminal nicotinamide clearance^6,7^ but now is known to exert multiple biological activity^8–10^. Our recent epidemiological study revealed that serum me-NAM was associated with obesity and diabetes in Chinese population^11^. In addition, our case-control study also suggested that serum me-NAM was associated with the presence and severity of coronary artery disease in Chinese patients^12^. Because obesity, diabetes and coronary artery disease were independent risk factors for the development of HF^13–15^, and left ventricular systolic dysfunction (LVSD) underlay most cases of HF but was often asymptomatic before its development^16^, we hypothesize that increased serum me-NAM levels might be associated with LVSD. Therefore, the present study aims to investigate the relationship between serum me-NAM levels and left ventricular structure and function in Chinese patients.

## Results

### Characteristics of the Study Participants

The 265 participants (mean age 65.8 ± 10.6 years) included 145 men (54.7%), 188 had hypertension (70.9%), and 80 had type 2 diabetes mellitus (30.2%). Table 1 summarizes the characteristics of the study participants categoried by serum me-NAM in tertiles. Total/HDL cholesterol ratio, and the prevalence of diabetes and proportion of antihyperglycemic treatment increased, while serum HDL cholesterol decreased across increasing tertiles of serum me-NAM (*P* ≤ 0.03, Table 1). Among echocardiographic indices, LVMI, and LVIDd, and the prevalence of LVH and LVSD significantly increased, while EF decreased across increasing tertiles of serum me-NAM (*P* ≤ 0.03, Table 1).

**Table 1:** Characteristics of the Study Subjects by Tertile Distribution of Serum N^1^-methylnicotinamide.

Characteristics	Tertile 1 (n = 88)	Tertile 2 (n = 88)	Tertile 3 (n = 89)	*P* for ANOVA
N^1^-methylnicotinamide, ng/ml	3.28 (2.22–4.03)	6.79 (5.73–8.00)^†^	12.17 (11.14–16.55)^‡^	<0.001
Male, n (%)	51 (58.0)	46 (52.3)	48 (53.9)	0.74
Current smoking, n (%)	25 (28.4)	20 (22.7)	24 (27.0)	0.67
Alcohol intake, n (%)	17 (19.3)	18 (20.5)	19 (21.4)	0.95
Hypertension, n (%)	64 (72.7)	66 (75.0)	58 (65.2)	0.32
Diabetes mellitus, n (%)	17 (19.3)	27 (30.7)	36 (40.5)^†^	0.009
Coronary artery disease, n (%)	46 (52.3)	60 (68.2)*	16 (82.0)^‡^	<0.001
Antihypertensive treatment, n (%)	57 (64.8)	58 (65.9)	53 (59.6)	0.65
Antihyperglycemic treatment, n (%)	14 (15.9)	21 (23.9)	29 (32.6)^†^	0.03
Age, years	65.0 ± 9.0	64.9 ± 10.4	67.5 ± 12.0	0.19
Body mass index, kg/m^2^	24.2 ± 3.4	25.1 ± 3.3	25.3 ± 3.4	0.10
Systolic blood pressure, mmHg	138.4 ± 18.0	137.0 ± 17.1	135.0 ± 20.4	0.47
Diastolic blood pressure, mmHg	82.9 ± 11.4	81.9 ± 10.7	80.5 ± 13.2	0.38
Heart rate, beats per minute	67.3 ± 9.8	67.9 ± 9.7	71.0 ± 12.1	0.10
Total cholesterol, mmol/L	4.08 ± 0.87	4.07 ± 0.90	4.16 ± 1.45	0.84
HDL cholesterol, mmol/L	1.26 ± 0.33	1.16 ± 0.2*	1.11 ± 0.3^‡^	0.002
Total/HDL cholesterol ratio	3.36 ± 0.83	3.61 ± 1.06	3.90 ± 1.33^†^	0.005
Fasting plasma glucose, mmol/L	5.52 ± 1.36	5.80 ± 1.59	6.03 ± 2.02	0.13
γ-glutamyltransferase, units/L	21.5 (14.5–30.5)	19.5 (13.0–34.6)	22.0 (15.0–43.0)	0.17
Serum creatinine, μmol/L	79.8 ± 24.7	81.7 ± 27.2	89.0 ± 28.6	0.11
eGFR, ml/(min• 1.73 m^2^)	81.4 ± 17.0	79.5 ± 17.4	74.8 ± 22.6	0.06
Left ventricular structure				
Left ventricular mass index, g/m^2^	93.8 ± 22.1	92.7 ± 23.7	101.8 ± 29.2*	0.03
Hypertrophy, n (%)	10 (11.4)	10 (11.4)	21 (23.6)*	0.03
Left ventricular end-diastolic diameter, cm	4.68 ± 0.45	4.74 ± 0.57	4.93 ± 0.79^†^	0.02
Relative wall thickness	0.39 ± 0.06	0.38 ± 0.06	0.38 ± 0.08	0.33
Systolic function				
Left ventricular ejection fraction, %	62.3 ± 4.3	61.1 ± 8.2	58.3 ± 11.1^†^	0.005
Preload recruitable stroke work, g/m^2^	86.6 ± 13.7	85.7 ± 14.6	78.6 ± 16.9^‡^	<0.001
Left ventricular systolic dysfunction, n (%)	1 (1.1)	8 (9.1)	15 (16.9)^‡^	0.001
Diastolic function				
E peak, cm/s	69.3 ± 21.1	69.2 ± 19.6	67.1 ± 18.9	0.75
E/A ratio	0.86 ± 0.28	0.86 ± 0.30	0.84 ± 0.36	0.88

Data are mean ± standard deviation, median with interquartile range in parenthesis, or number with percentage in parenthesis. HDL, indicates high-density lipoprotein; eGFR, estimated glomerular filtration rate. For definitions of hypertension, diabetes, coronary artery disease, left ventricular hypertrophy, and left ventricular systolic dysfunction, see Methods. *P < 0.05; ^†^*P* < 0.01; ^‡^*P* < 0.001, vs Tertile 1.

### Association of N^1^-methylnicotinamide With Left Ventricular Structural and Functional Indices

We next investigated the relationship of serum me-NAM levels with left ventricular structural and functional Indices. Serum me-NAM concentrations were positively associated with left ventricular end-diastolic dimension (r = 0.15; *P* = 0.01) and negatively associated with EF (r = −0.23; *P* < 0.001) and preload recruitable stroke work (PRSW) (r = −0.20; *P* = 0.002) but not associated with left ventricular diastolic function (r = −0.02; *P* ≥ 0.73). However, only associations of serum me-NAM with EF and PRSW remained statistically significant (*P* ≤ 0.02, Table 2) after adjustments for age, sex, body mass index, systolic blood pressure, current smoking and alcohol intake, hypertension, diabetes, use of antihypertensive and antihyperglycemic drugs, fasting plasma glucose, total to HDL cholesterol ratio, and estimated glomerular filtration rate. Separating all subjects into men and women did not materially change the results. (Supplemental Table S1).

**Table 2:** Simple and multivariate adjusted correlations between N^1^-methylnicotinamide and left ventricular structure and function.

	Serum N^1^-methylnicotinamide* (ng/ml, Log)			
	r	*P*	Partial r	*P*
Left ventricular mass index, g/m^2^	0.08	0.19	0.07	0.26
Left ventricular end-diastolic diameter, cm	0.15	0.01	0.13	0.06
Relative wall thickness	−0.12	0.05	−0.13	0.05
Left ventricular ejection fraction, %	−0.23	<0.001	−0.19	0.003
Preload recruitable stroke work, g/m^2^	−0.20	0.002	−0.15	0.02
E peak, cm/s	−0.02	0.73	−0.08	0.23
E/A ratio	−0.02	0.75	−0.04	0.55

^*^Log-transformed variable. We performed simple and multivariate adjusted correlation analyses of relationship between N^1^-methylnicotinamide and left ventricular structure and function. For multivariate adjusted correlation, age, sex, body mass index, systolic blood pressure, current smoking and alcohol intake, hypertension, diabetes, coronary artery disease, use of antihypertensive and antihyperglycemic drugs, fasting plasma glucose, total to high density lipoprotein cholesterol ratio and estimated glomerular filtration rate were adjusted.

### Association of N^1^-methylnicotinamide With Presence of LVH and LVSD

Because men and women had similar correlates of serum me-NAM and measurements of left ventricular structure and function, we combined men and women in further logistic regression analyses. In simple and multiple logistic regression analyses, serum me-NAM was not significantly associated with the prevalence of LVH (*P* ≥ 0.054, Table 3). However, compared to those in the lowest tertile of serum me-NAM levels, subjects in the highest tertile were associated with an increased odds ratio of LVSD both before and after adjustment for potential confounders (i.e., age, sex, body mass index, systolic blood pressure, current smoking and alcohol intake, hypertension, diabetes, use of antihypertensive and antihyperglycemic drugs, fasting plasma glucose, total to HDL cholesterol ratio, and estimated glomerular filtration rate) (*P* ≤ 0.026, Table 3). Similar results were obtained by logistic regression using log-transformed me-NAM as a continuous variable (*P* ≤ 0.02, Table 4).

**Table 3:** Associations of serum N^1^-methylnicotinamide concentration tertiles with left ventricular hypertrophy and systolic dysfunction.

	Serum N^1^-methylnicotinamide, ng/ml (Tertile 2 vs. Tertile 1)		Serum N^1^-methylnicotinamide, ng/ml (Tertile 3 vs. Tertile 1)	
	Odds ratio (95% CI)	*P*	Odds ratio (95% CI)	*P*
**Left ventricular hypertrophy**				
Crude model	0.82 (0.32–2.08)	0.67	2.19 (0.99–4.87)	0.054
Adjusted model	0.86 (0.31–2.33)	0.76	1.97 (0.76–5.11)	0.17
**Left ventricular systolic dysfunction**				
Crude model	3.81 (0.77–18.86)	0.10	8.82 (1.95–39.82)	0.005
Adjusted model	3.37 (0.62–18.25)	0.16	6.80 (1.26–36.72)	0.026

In the adjusted model, odds ratio (95% CI) were adjusted for age, sex, body mass index, systolic blood pressure, current smoking and alcohol intake, hypertension, diabetes, coronary artery disease, use of antihypertensive and antihyperglycemic drugs, fasting plasma glucose, total to high density lipoprotein cholesterol ratio and estimated glomerular filtration rate.

**Table 4:** Associations of serum N^1^-methylnicotinamide concentrations with left ventricular hypertrophy and systolic dysfunction.

	Serum N^1^-methylnicotinamide (+1 ng/ml, Log)	
	Odds ratio (95% CI)	*P*
**Left ventricular hypertrophy**		
Crude model	2.53 (0.90–7.14)	0.08
Adjusted model	1.69 (0.53–5.41)	0.38
**Left ventricular systolic dysfunction**		
Crude model	14.45 (3.35–62.28)	<0.001
Adjusted model	9.48 (1.41–63.48)	0.02

In the adjusted model, odds ratio (95% CI) were adjusted for age, sex, body mass index, systolic blood pressure, current smoking and alcohol intake, hypertension, diabetes, coronary artery disease, use of antihypertensive and antihyperglycemic drugs, fasting plasma glucose, total to high density lipoprotein cholesterol ratio and estimated glomerular filtration rate.

## Discussion

One of the key findings of this study is that serum me-NAM, an indicator of NNMT activity, is negatively associated with left ventricular EF and PRSW, and accordingly positively associated with the prevalence of LVSD, independent of conventional risk factors. Among 265 Chinese subjects in our study, compared with those in the lowest tertile of serum me-NAM, subjects in the highest are associated with an approximate 7-fold increase in the risk for LVSD. As LVSD is predictive of cardiovascular events^17,18^, our findings therefore might have potential clinical implications for cardiovascular events prevention through inhibiting me-NAM formation.

To the best of our knowledge, our study is the first that has demonstrated significant association of serum me-NAM concentration with reduced left ventricular EF and PRSW and systolic dysfunction. Two previous studies with smaller sample sizes examined me-NAM levels in HF. The first untargeted metabolomic analyses revealed low urinary me-NAM in humans with HF^19^, whereas the second study showed that urinary me-NAM levels are lower in HF compared with healthy controls but they did not find significant association between urinary me-NAM levels and left ventricular EF^20^. These findings partly support our results that serum me-NAM levels are not only negatively associated with left ventricular EF and PRSW, but also with the risk of LVSD. Me-NAM is an endogenous metabolite of nicotinamide, which is cleared by the kidney, without being reabsorbed to a significant extent. Thus, its clearance in the kidney reflects the renal plasma flow^21^. Urinary me-NAM concentration is lower in HF^19,20^ because glomerular filtration rate is often reduced in this condition. Our explanation on the significant positive association of serum me-NAM with prevalence of LVSD can be inferred from that higher serum me-NAM levels due to its slower renal clearance because of reduced renal plasma flow with HF. Indeed, serum me-NAM was negatively associated with eGFR (r = −0.17; *P* = 0.007), independent of other confounders, in our study. The discrepancy between our findings and the results of a previous study^20^ might be explained by the difference in the sample size between the two studies. In 46 HF patients and 32 control subjects, Chung JH *et al*.^20^ measured urinary me-NAM by nuclear magnetic resonance and reported lower levels of urinary me-NAM in HF, which is in line with our results. However, in this previous study, urinary me-NAM concentration was not associated with left ventricular EF. This previous study apparently has insufficient power to show association of the size as observed in our study.

The mechanism underlying the elevation of serum me-NAM in LVSD patients has not been clear. However, this observation is consistent with our recent findings on the association of serum me-NAM with obesity and diabetes in a population study^11^, and with coronary artery disease in a case-control study^12^. Indeed, obesity, diabetes and coronary artery disease are closely related to LVSD^22,23^. In addition, higher serum me-NAM can induce imbalance in the NAD^+^/NADH redox couple, which contributes to the development of HF because interventions normalizing NAD^+^/NADH imbalance convey benefits for the failing hearts^24^. Further studies are needed to delineate the mechanisms of me-NAM in the development of HF.

Our study should be interpreted within the context of its limitations. The cross-sectional design does not allow causal inference. In addition, we only investigated me-NAM levels in Chinese, and therefore our findings need to be confirmed in other ethnicities in future. Finally, the measures used for diastolic function have some limitations. Future studies including data of comprehensive parameters of diastolic function are warranted to further explore the relationship between serum me-NAM and LV diastolic function.

In summary, our study shows significantly negative associations of serum me-NAM with left ventricular EF and PRSW, and accordingly positive associations with the prevalence of LVSD in Chinese patients. If confirmed also in prospective studies with hard cardiovascular outcomes, such as HF, our findings may have potential clinical implications. More importantly, further experimental investigation should be performed to delineate whether changes in serum me-NAM by inhibiting NNMT activity would improve left ventricular systolic function, and subsequently provide benefits to patients with HF.

## Methods

### Study Population

Subjects were recruited in the framework of a case-control study in suspected coronary artery disease (CAD), as reported previously^12^. From April 2015 to July 2015, we studied 342 consecutive patients who were suspected of having CAD with chest pain and were referred for diagnostic coronary angiography to Department of Cardiology, Jiangsu Province Hospital of Traditional Chinese Medicine, Nanjing, China. We excluded 77 subjects from the present analyses because of inadequate blood samples for me-NAM measurements (n = 3) or missing information on echocardiographic data (n = 68), plasma glucose (n = 2), serum lipids (n = 3) or serum creatinine (n = 1). Thus, the total number of subjects in the present analyses was 265. All subjects gave written informed consent. The study protocol was approved by the ethic committee of Jiangsu Province Hospital of Traditional Chinese Medicine. All procedures were performed in accordance with the guidelines of the Helsinki Declaration.

### Anthropometric and Biochemical Measurements

Sociodemographics, medical history, smoking and drinking habits, and the use of medications were documented with a standardized questionnaire. Body weight, body height, and blood pressure were measured by an experienced nurse. BMI was calculated as weight in kilograms divided by height in meters squared. Three sitting blood pressure measurements taken consecutively at 5-minutes intervals using a validated electronic device (Omron HEM 7130, Japan) were averaged for analysis. Venous blood samples were taken after overnight fasting for the measurement of plasma glucose, serum creatinine, total cholesterol, high-density lipoprotein (HDL) cholesterol, low-density lipoprotein (LDL) cholesterol, triglycerides (TG), and γ-glutamyltransferase (GGT). Serum samples were subsequently stored in aliquots at −80 °C for measurements of me-NAM.

As described previously^11,12^, serum me-NAM concentrations were determined by liquid chromatography with tandem mass spectrometry (LC/MS/MS, Agilent 6430 Triple Quad LC/MS, USA) in the Clinical Pharmacology Laboratory, Jiangsu Province Hospital of Traditional Chinese Medicine. The intra- and inter-assay coefficients of variance were 3.69% and 4.95% for me-NAM, respectively. The analytic sensitivity of the assays for measuring me-NAM was 2.5 ng/ml.

Hypertension was defined as a blood pressure of at least 140 mmHg systolic or 90 mmHg diastolic, or as the use of antihypertensive drugs. Diabetes mellitus was defined as a fasting plasma glucose of at least 7.0 mmol/L or as the use of anti-diabetic agents.

### Echocardiographic measurements

M-mode and 2-dimensional transthoracic echocardiography were performed with the GE Vivid E9 system (General Electric Healthcare, Milwaukee, WI, USA), as recommended by the American Society of Echocardiography (ASE). Measurements for M-mode guided calculation of left ventricular mass (LVM) were taken in the parasternal short-axis view. Left ventricular internal dimension (LVIDd) and septal (SWTd) and posterior wall thickness (PWTd) at end-diastole were evaluated. LVM was calculated by the ASE recommended formula^25^: LVM (g) = 0.8 × {1.04 × [(LVIDd + PWTd + SWTd)^3^ − (LVIDd)^3^]} + 0.6, and standardized to body size by dividing the LVM by body surface area, as left ventricular mass index (LVMI). Left ventricular hypertrophy (LVH) was defined as a LVMI > 125 g/m^2^ in men or a LVMI > 110 g/m^2^ in women, respectively^26^. Relative wall thickness (RWT) was calculated by the formula: (2 × PWTd)/LVIDd, and also used as a measure of left ventricular geometry^27^. Left ventricular EF and PRSW were used as indices of left ventricular systolic function^28^. Left ventricular EF was assessed by two-dimensional echocardiography using the modified Simpson’s method. PRSW was determined by the single-beat method of Karunanithi and Feneley^29^ and Lee *et al*.^30^. Left ventricular systolic dysfunction (LVSD) was defined as an EF less than 50%. The early diastolic peak flow (E) and atrial peak flow (A) were recorded by the pulse wave Doppler, and the E/A ratio was calculated as indicator of left ventricular diastolic function. All the echocardiographic measurements were performed by two experienced sonographers (Anxia He and Chao Chen) who were blinded to the results of the me-NAM assay as well as to details of clinical details.

### Statistical Methods

Statistical analyses were performed using SAS software, version 9.2 (SAS institute, Cary, NC, USA). Data were presented as mean ± SD or median (25th and 75th percentiles) for continuous variables, or as percentage for categorical variables. Departure from normality was tested by the Shapiro–Wilk’s statistic. Serum me-NAM concentrations were not normally distributed and were, therefore, logarithmically transformed for statistical analyses. Means and proportions were compared with the Student *t* test and Fisher’s exact test, respectively. Relationships among me-NAM, and left ventricular structure and function indices were examined by calculation of partial correlation coefficients. Multiple logistic regression analyses were employed to evaluate the odds ratios (OR) and 95% confidence intervals (CI) of having LVH and LVSD for higher tertiles of me-NAM compared with the lowest tertile or for each 1 unit increase in the logarithmically transformed me-NAM. A two-sided value of *P* < 0.05 was considered statistically significant.

### Data availability

The datasets generated and analysed during the current study are available from the corresponding author on reasonable request.

## Electronic supplementary material

Table S1
