# Supplementary material for: Serum N1-methylnicotinamide is Associated with Left Ventricular Systolic Dysfunction in Chinese
Source: Sci Rep. 2018 Jun 5;8:8581. doi: 10.1038/s41598-018-26956-7 (PMC5988810; doi:10.1038/s41598-018-26956-7)
Supplement: Supplementary file 1 — Table S1 [file 41598_2018_26956_MOESM1_ESM.doc]

Serum N^1^-methylnicotinamide is Associated with Left Ventricular

Systolic Dysfunction in Chinese

Ming Liu MD PhD^1,2,†^, Anxia He MD^3,†^, Jihong Chu MD^4,†^, Chao Chen MD^3^,

Siqi Zhang MD^1^, Yun He MD^1^, Weiwei Tao MD^3^, Meijuan Lu MD^3^,

Mulian Hua MD^1^, Wenzheng Ju MD PhD^4^, Zhuyuan Fang MD PhD^1,2,*^

^1^Institute of Hypertension, Jiangsu Province Hospital of Traditional Chinese Medicine, The Affiliated Hospital of Nanjing University of Chinese Medicine, Nanjing, Jiangsu, China;

^2^Department of Cardiology, Jiangsu Province Hospital of Traditional Chinese Medicine, The Affiliated Hospital of Nanjing University of Chinese Medicine, Nanjing, Jiangsu, China;

^3^Department of Echocardiography, Jiangsu Province Hospital of Traditional Chinese Medicine, The Affiliated Hospital of Nanjing University of Chinese Medicine, Nanjing, Jiangsu, China;

^4^Department of Clinical Pharmacology, Jiangsu Province Hospital of Traditional Chinese Medicine, The Affiliated Hospital of Nanjing University of Chinese Medicine, Nanjing, Jiangsu, China.

**Correspondence to:**

Zhuyuan Fang, MD, PHD,
Institute of Hypertension, Department of Cardiology, Jiangsu Province Hospital of TCM
155 Hanzhong Road,
Nanjing, Jiangsu Province210029, China,

Tel: +86-25-86619893,
Fax: +86-25-86618942,
E-mail:jsszyyfzy@163.com

**Supplemental Table S1. Simple and multivariate adjusted correlations between N^1^-methylnicotinamide and left ventricular structure and function by sex**

|  | **Serum *N^1^*-methylnicotinamide*** **(ng/ml, Log)** | | | | | | | | |
| --- | --- | --- | --- | --- | --- | --- | --- | --- | --- |
|  | **Men (n=145)** | | | |  | **Women (n=120)** | | | |
|  | ***r*** | ***P*** | ***Partial r*** | ***P*** |  | ***r*** | ***P*** | ***Partial r*** | ***P*** |
| Left ventricular mass index, g/m^2^ | 0.07 | 0.40 | 0.08 | 0.34 |  | 0.12 | 0.19 | 0.12 | 0.21 |
| Left ventricular end-diastolic diameter, cm | 0.15 | 0.07 | 0.13 | 0.15 |  | 0.22 | 0.02 | 0.17 | 0.05 |
| Relative wall thickness | −0.15 | 0.07 | −0.15 | 0.09 |  | −0.08 | 0.36 | −0.07 | 0.45 |
| Left ventricular ejection fraction, % | −0.28 | <0.001 | −0.22 | 0.01 |  | −0.21 | 0.02 | −0.20 | 0.04 |
| E peak, cm/s | −0.06 | 0.52 | −0.16 | 0.08 |  | −0.004 | 0.97 | −0.03 | 0.79 |
| E/A ratio | −0.09 | 0.19 | −0.13 | 0.16 |  | −0.07 | 0.44 | 0.08 | 0.44 |

*Log-transformed variable. We performed sex-specific simple and multivariate adjusted correlation analyses of relationship between me-NAM and left ventricular structure and function. For multivariate adjusted correlation, age, body mass index, systolic blood pressure, current smoking and alcohol intake, hypertension, diabetes, coronary artery disease, use of antihypertensive and antihyperglycemic drugs, fasting plasma glucose, total to high density lipoprotein cholesterol ratio and estimated glomerular filtration rate were adjusted.
